# Widely Targeted Metabolomics Analysis Reveals the Effect of Flooding Stress on the Synthesis of Flavonoids in *Chrysanthemum morifolium*

**DOI:** 10.3390/molecules24203695

**Published:** 2019-10-14

**Authors:** Tao Wang, Qingjun Zou, Qiaosheng Guo, Feng Yang, Liwei Wu, Wenyan Zhang

**Affiliations:** Institute of Chinese Medicinal Materials, Nanjing Agricultural University, Nanjing City, Jiangsu Province 210095, China; wt1344@njau.edu.cn (T.W.); 2018204046@njau.edu.cn (Q.Z.); 2018204047@njau.edu.cn (F.Y.); 14415117@njau.edu.cn (L.W.); 2017104121@njau.edu.cn (W.Z.)

**Keywords:** metabolomics, flavonoid biosynthesis, *Chrysanthemum morifolium*, flooding stress

## Abstract

*Chrysanthemum morifolium*. cv “Hangju” is an important medicinal material with many functions in China. Flavonoids as the main secondary metabolites are a major class of medicinal components in “Hangju” and its composition and content can change significantly after flooding. This study mimicked the flooding stress of “Hangju” during flower bud differentiation and detected its metabolites in different growth stages. From widely targeted metabolomics data, 661 metabolites were detected, of which 46 differential metabolites exist simultaneously in the different growth stages of “Hangju”. The top three types of the 46 differential metabolites were flavone C-glycosides, flavonol and flavone. Our results demonstrated that the accumulation of flavonoids in different growth stages of “Hangju” was different; however, quercetin, eriodictyol and most of the flavone C-glycosides were significantly enhanced in the two stages after flooding stress. The expression of key enzyme genes in the flavonoid synthesis pathway were determined using RT-qPCR, which verified the consistency of the expression levels of *CHI*, *F3H*, *DFR* and *ANS* with the content of the corresponding flavonoids. A regulatory network of flavonoid biosynthesis was established to illustrate that flooding stress can change the accumulation of flavonoids by affecting the expression of the corresponding key enzymes in the flavonoid synthesis pathway.

## 1. Introduction

*Chrysanthemum morifolium* Ramat. is one of the most important multi-purpose crops with ornamental value, medicinal value and edible value [1,2,3]. Dried capitulum of *C. morifolium*, Chrysanthemi Flos, is an important medicinal material in China, Japan, Korea and other countries in East Asia [4], which is effective for anemopyretic colds, headaches, and dim-sighted eyes [5]. Modern pharmacological research has shown that it has broad effects, such as antibacterial, antiviral, potent neuroprotective and anti-inflammatory activities and is cardiovascular protective [6,7,8,9,10]. Based on growing regions and processing methods, the main domestic varieties of medicinal *C. morifolium* are divided into “Hangju”, “Boju”, “Qiju”, “Gongju”, “Jiju”, “Chuju”, “Huaiju” and “Chuanju” [11,12] of which the “Hangju” type is found in the most cultivated areas and has the highest yield It is believed that the “Hangju” from the origin (Tongxiang County, Zhejiang Province, China) is considered to be a top-geoherb. The top-geoherbs used in China are prized for their high qualities and are grown in specified areas with specific environments [13].

As the demand for “Hangju” soared, the cultivated areas were greatly expanded from the origin. However, with the unordered introduction to the expanded area, the medicinal quality of “Hangju” declined rapidly. Earlier research found that the region of origin was more suitable for “Hangju” than the introduction areas on the aspects of drug properties and active index component, especially the content of flavonoids [14]. Flavonoids, such as anthocyanins, flavones and flavonols, are important secondary compounds in higher plants. Flavonoids are involved in many physiological roles, such as pollinator attraction in petals, protection against UVB light, interaction with microorganisms, antipathogenic phytoalexins, and fertility and germination of pollen [15,16,17,18]. In addition, flavonoids also function as antioxidants and potential anticancer agents in humans [16], as well as possessing antibacterial, anti-inflammatory, antiviral and cardiovascular activities [15,19,20]. Scientists have also shown that the flavonoid constituents luteolin and quercetin act as antineoplastic and anti-HIV agents [10].

Components of Chinese traditional medicine are usually secondary metabolites, and a large number of studies have shown that physical and chemical stress can increase the accumulation of secondary metabolites [13,21,22]. From comprehensive analysis of the environmental factors of all producing areas, the environmental difference between the introduced area and the origin is mainly reflected in the difference in annual precipitation. In the origin of Tongxiang, which is located in the summer typhoon area, the precipitation in August was significantly higher than in the other cultivated regions, leading the “Hangju” there to inevitably encounter flooding stress. Coincidentally, “Hangju” bud differentiation occurs during mid-to-late August, during which the medicinal substances are synthesized [23]. The change in soil properties caused by flooding is one of the important factors affecting the normal growth and development of plants, and it is also one of the main factors that restricts the distribution of species and agricultural productivity. Of course, flooding stress is also a powerful force for the adaptive evolution of plants [24]. Therefore, this research is based on drowning the “Hangju” during the flower bud differentiation stage to understand the expression of key enzyme genes and the content of flavonoids under flooding stress.

## 2. Results

### 2.1. Differences in Total Flavonoids and Anthocyanins Contents in “Hangju” After Flooding Stress

We simulated the natural flooding situation similar to the original area of “Hangju” and carried out the flooding treatment of the potted “Hangju” in the flower bud differentiation period for three consecutive days. After the stress was relieved, we harvested its capitula in the bud stage (BS) and the flower bloom stage (FBS) to measure the total flavonoids and anthocyanins. The results showed that the total flavonoids in the treatment group was significantly higher than the total in the control group during the two periods. In contrast, the content of anthocyanins in the treatment group was significantly lower than in the control group.

### 2.2. Metabolite Profiling of “Hangju” in Different Growth Stages After Flooding Stress Treatment

**Figure 1:**
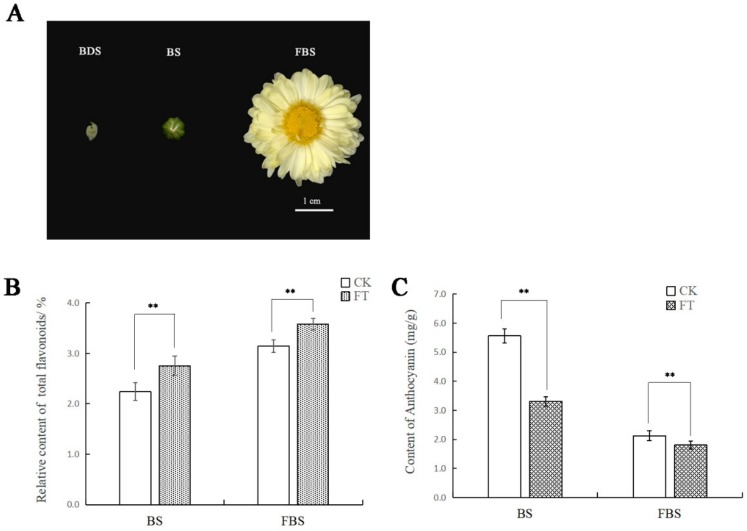
Content of total flavonoids and anthocyanins in different stages of “Hangju”. (**A**) Morphological characteristics of “Hangju” capitula in different growth stages: flower bud differentiation stage (BDS), bud stage (BS) and flower bloom stage (FBS). Content of total flavonoids (**B**) and anthocyanin (**C**) in different stages after flooding stress. Data represent mean values ± SD of three independent measurements. ** indicates a significant difference at *p* < 0.01.

**Figure 2:**
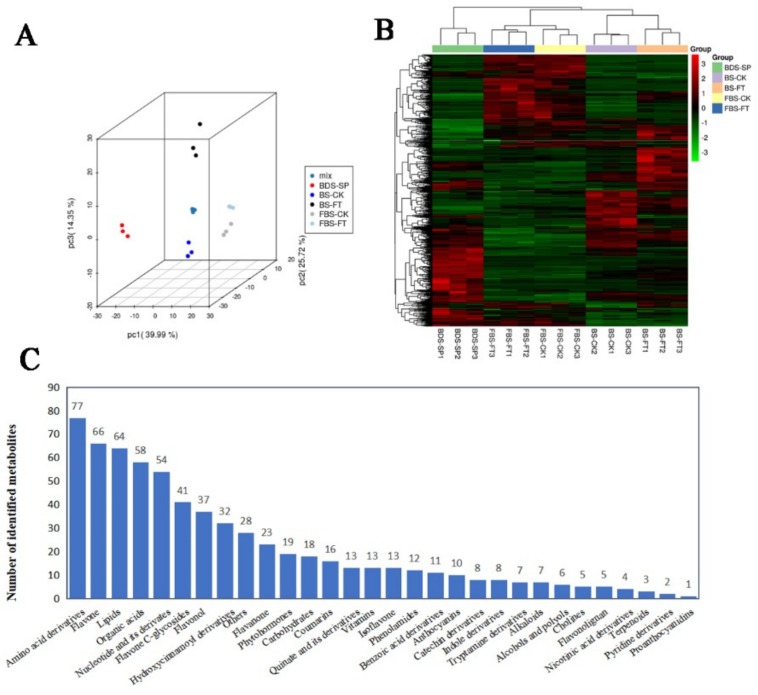
Quality control analysis and metabolite identification of all samples. (**A**) Score plot of principal components analysis for all samples based on mass spectrometry data. Mix refers to the mixing of all samples as a quality control sample. SP refers to the starting point of flooding; CK refers to the control group; FT refers to the flooding treatment group. (**B**) Heat map of all samples by hierarchical cluster analysis. (**C**) Classes and quantity of identified metabolites.

In order to explore the effects of flooding stress on the metabolism of “Hangju” during flower bud differentiation, we collected 15 samples from 3 different periods (Figure 1A) for extensive targeted metabolome analysis. Based on the self-built database (Metware Database) [25], the metabolites were characterized according to the secondary spectrum information. Metabolite quantification was performed using multiple reaction monitoring mode analysis with triple quadrupole mass spectrometry [26]. In this study, 661 metabolites were detected (Table S1), of which 286 were identified in negative-ionization mode and 375 in positive-ionization mode. Principal component analysis was performed on samples, including quality control samples (mix of all samples), to provide an initial understanding of the overall metabolic differences between groups of samples and the magnitude of variability between samples within the group. The results found that the first three principal components explained 39.99%, 25.72% and 14.35% of the metabolic variances for all samples (Figure 2A). According to the principal components analysis (PCA) score map, the metabolites of “Hangju” in different growth stages can be clearly distinguished, which proves that the different growth stages of “Hangju” have large differences in metabolite composition, and the samples in the group are aggregated together with less variation. At the same time, the data was normalized and all samples were analyzed using a clustering heat map (Figure 2B). The results showed that the repeatability between groups was consistent. All metabolites were clustered into three main categories, and each cluster had significant differences in the growth stages of “Hangju”. The metabolite categories are shown in Figure 2C, with the top five in the rankings being amino acid derivatives, flavone, lipids, organic acids, and nucleotide and its derivatives. Two hundred and eighty of all measured metabolites were annotated from the Kyoto Encyclopedia of Genes and Genomes (KEGG, https://www.kegg.jp) database (Table S2).

### 2.3. Analysis of Differential Metabolites in “Hangju” After Flooding Stress

**Figure 3:**
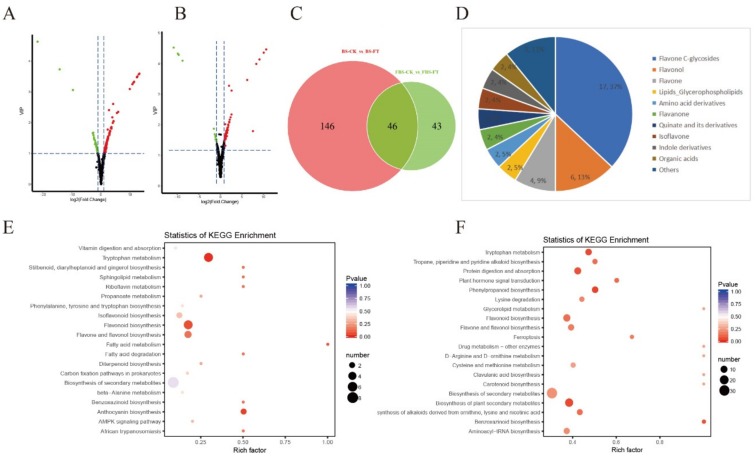
The differential metabolites analysis in BS and FBS, respectively. The volcano plots show the differential metabolites expression levels after flooding stress in BS (**A**) and FBS (**B**). Green dots represent down-regulated differentially expressed metabolites; red dots represent up-regulated differentially expressed metabolites; and black dots represent non-differentially expressed metabolites. (**C**) The Venn diagram shows the overlapping and specific differential metabolites from groups of BS-CK vs. BS-FT and FBS-CK vs. FBS-FT. (**D**) The pie chart shows classes and quantity of 46 overlapping metabolites from Figure 3C. Enrichment of differential metabolites to distinct KEGG pathways from BS (**E**) and FBS (**F**). The abscissa indicates the rich factor of each pathway, the ordinate is the name of pathway, and the color of the point reflects the P value. The size of the dots represents the number of differential metabolites enriched.

In this study, differential metabolites were screened by combining the fold change and the variable importance in project (VIP) value, which come from orthogonal partial least squares discriminant analysis (OPLS-DA) model. OPLS-DA combines orthogonal signal correction (OSC) and partial least squares discriminant analysis (PLS-DA) methods to decompose X-matrix information into two types of information that are related and unrelated to Y, and then remove irrelevant differences to filter for difference variables. High predictability (Q^2^) and strong goodness of fit (R^2^X, R^2^Y) of the PLS-DA models were observed in the comparison between BS-FT and BS-CK (Q^2^ = 0.992, R^2^X = 0.854, R^2^Y = 0.99), as well as between FBS-FT and FBS-CK (Q^2^ = 0.975, R^2^X = 0.703, R^2^Y = 0.999) (Figure S1). To gain more insight into the metabolic differences between BS-FT and BS-CK, and FBS-FT and FBS-CK, respectively, differential metabolites screening was performed among all 661 metabolites identified according to the fold-change and the variables determined to be important in the projection (VIP) scores. A fold-change score ≥ 2 or ≤ 0.5 among the metabolites with a VIP value ≥ 1 was used as an identification criterion. The screening results are illustrated using volcano plots (Figure 3A,B). The results showed that there were 192 (up-regulated metabolites were 140) and 89 (up-regulated metabolites were 75) significant metabolites in the BS and FBS periods, respectively. The evidence highly suggests that the influence of flooding stress in the BS on the metabolism of “Hangju” is greater than that in the FBS. Then, we mapped the Venn diagrams of the differential metabolites from BS and FBS and found that there were 46 concurrent differential metabolites in both stage (Figure 3C and Table S3). The top three types of metabolites were flavone C-glycosides, flavonol and flavone (Figure 3D).

The differential metabolites interacted in the organism to form different pathways, and the differential metabolites were annotated and displayed using the KEGG database. The results showed that the pathway with the largest number of differential metabolites was biosynthesis of secondary metabolites both in the BS and FBS stages (Figure 3E,F). Then, according to the number of differential metabolites and the degree of enrichment, the order of tryptophan metabolism, flavonoid biosynthesis and flavone and flavonol biosynthesis was followed. Plant secondary metabolites are non-essential small molecular organic compounds that are produced by secondary metabolism, which usually possess bioactivity.

### 2.4. Analysis of Differential Metabolites in “Hangju” After Flooding Stress

**Figure 4:**
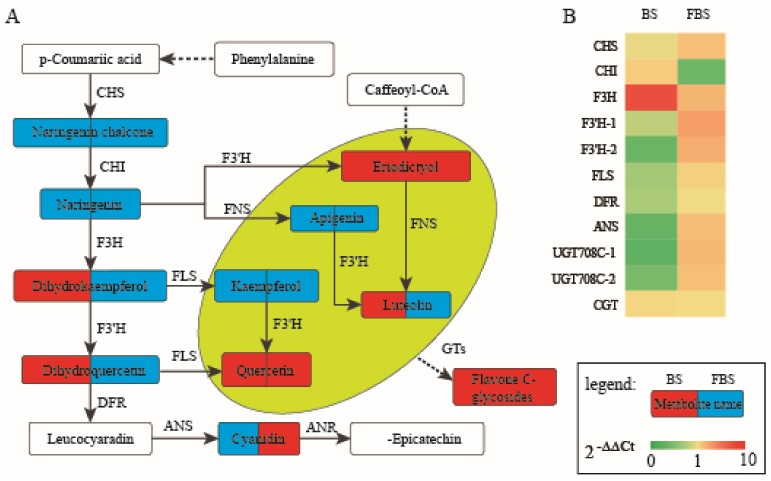
Regulatory network of flavonoid biosynthesis after flooding stress in bud and flower bloom stages. (**A**) The synthesis pathway of flavonoids metabolites. The left half of the rectangle represents the change of metabolites in the BS, and the right half represents the change of metabolites in the FBS. Blue means no change of metabolites, red means increase, and white means not detected. (**B**) The fold change of key enzyme genes after flooding stress in two stages. CHS, chalcone synthase; CHI, chalcone isomerase; F3H, flavanone 3-hydroxylase; F3′H, flavonoid 3’-hydroxylase; FLS, flavonol synthase; DFR, flavanone 4-reductase; FNS, flavone synthase; ANS, anthocyanidin synthase; ANR, anthocyanidin reductase; GT, glycosyltransferase.

The flavonoids are considered to be the main medicinal components of medicinal chrysanthemums, and the above results indicated that flooding stress has a great influence on the accumulation of flavonoid metabolites in “Hangju”. Based on the above, flavonoid metabolites were mapped to the KEGG database in order to obtain detailed pathway information. We found that, while the accumulation of flavonoids varied in the different growth stages of “Hangju”, quercetin, eriodictyol and most of flavone C-glycosides were significantly enhanced in both stages. In order to better understand the relationship between metabolites and genes in flavonoid biosynthesis, the key enzyme genes in the flavonoid synthesis pathway were screened from the existing transcriptome database for qPCR verification, and the results are shown in Figure 4. Among these key enzyme genes, the expression trends of F3H and ANS were consistent with the trends of changes in downstream metabolite content, while the expression of F3’H and FLS appeared to be complicated by the involvement of multiple branches. However, there was no significant correlation between the expression levels of the three glycosyltransferase genes and the content of flavone C-glycosides, and the regulation of these genes needs further study.

## 3. Discussion

### 3.1. Metabolome Analysis to Evaluate the Quality of Medicinal Plants

Metabolites as the material basis of the phenotype of organisms can help us understand biological processes and mechanisms more intuitively and effectively. Therefore, metabolomics is used not only to study the accumulation and distribution of metabolites in organisms, but also the relationship to the phenotype and heredity [27,28,29,30]. With the development of metabolite profiling technologies suitable for large-scale measurement, metabolomics is now playing a significant role in both fundamental plant biology and applied biotechnology [31,32,33]. There are currently three main metabolomics technologies: targeted metabolomics, untargeted metabolomics, and widely targeted metabolomics. Among them, widely targeted metabolomics can simultaneously quantify hundreds of known metabolites and nearly 1000 known and unknown metabolites using the innate Q TRAP mass spectrometry in multiple reaction monitoring (MRM) mode, which enables high-throughput, highly sensitive, and widely covered metabolite detection. One study compared the metabolite differences between two sesame seeds using widely targeted metabolomics and screened biomarkers that significantly related to the functions described in Chinese medicine [34]. They used combined transcriptome and metabolome analysis methods to explore the molecular mechanism of the synthesis of C-glycosylated flavonoids in the synthetic pathway of puerarin, the main medicinal component in *Pueraria lobate* [35]. Another study compared the differential metabolites of two varieties of *Ginkgo biloba* and combined them with transcriptome to reveal a new flow of phenylpropanoid metabolites in *Ginkgo biloba* [36]. The biosynthetic pathways of many traditional Chinese medicine ingredients, as well as their accumulation in different growth stages and tissue differences, are still unclear. Widely targeted metabolomics is an effective method for evaluating the quality of traditional Chinese medicines because it can accurately characterize and quantify a large number of secondary metabolites. It is combined with transcriptome sequencing technology to perform co-expression and joint analysis, which provides a powerful approach to candidate functional genes and construct metabolic networks.

### 3.2. Relationship between Flooding Stress and Medicinal Quality of “Hangju”

The living environment of plants is not always ideal. Environmental stress, also called adversity, is experienced throughout the whole process of plant growth. The active ingredients of medicinal plants are generally secondary metabolites, and the formation of these secondary metabolites is dependent on the stimulation of plants by stress [22]. The hormesis theory states that certain limits of harmful stimuli or unfavorable factors activate the stress-adaptive response of the organism, forming a protective mechanism that can counter severe or even devastating damage [37,38]. Different from the cultivation of food crops, the cultivation of medicinal plants should incorporate the accumulation of secondary metabolites into the production target [39]. Therefore, medicinal plant cultivation should not choose the most suitable cultivation environment for the accumulation of primary metabolites, but one that has some environmental stress factors to achieve as high-quality production as possible. Flooding is a common environmental stress, which alters the original growth environment and conditions experienced by plants, greatly affecting plant growth and physiological rhythms [40]. Guo et al. (2005) found that the content of volatile oil in *Atractylodes lancea* increased significantly with the increase of rainfall [41]. Martin et al. (2002) found that the total phenolic content in the leaves of *Solanum carolinense* under normal cultivation conditions was much lower than that under drought stress [42]. The results of this study indicated that flooding stress could significantly increase the total flavonoid content of “Hangju”, and also affect the secondary metabolic biosynthesis. The synthesis of flavonol and anthocyanin has a competitive relationship. Dihydroflavonol is the common upstream substrate of the flavonol and anthocyanin pathways, which are competed for by DFR and FLS respectively, the two key enzymes in their synthetic pathway [43]. The decrease in the expression of DFR may be one of the reasons for the decrease in anthocyanin content. At the same time, it was found that flooding stress caused significant differences in the contents of flavone C-glycosides, flavonol and flavone. As often claimed, flavonoids exhibit a variety of biological activities, not only to plants, which produce these compounds, but also to animals, which intake flavonoids in their diet [44,45,46]. This result is consistent with the results of previous studies, which means that flooding stress does cause the increase of flavonoids in “Hangju” to resist stress, and this phenomenon is conducive to the formation of the medicinal quality of “Hangju”.

## 4. Materials and Methods

### 4.1. Plant Material and Growth Condition

The plant material, *C. morifolium* cv. “Hangju”, obtained by tissue culture from the same parent strain, was planted in the germplasm repository of Nanjing Agricultural University. We adopted the method of potting in 21 cm-inner diameter, 17-cm-tall flowerpots. Each pot contained one “Hangju” plant. Plants with similar size and growth conditions were selected and divided into two groups—one experimental and one controlled, with 15 plants in each group. We set 3 biological replicates separately, each containing 5 plants.

### 4.2. Flooding Treatment and Sampling Method

On 25th August, the period of the flower bud differentiation stage was determined by morphological anatomy. We placed the potted “Hangju” plants into bigger pots without holes and added water to submerge the soil. The soil in the pot was completely submerged by water and it was ensured that the water was 2 cm above the soil surface. Then, we used a soil moisture meter (TDR 150, Spectrum Technologies Inc., Aurora, IL, USA) to detect soil volumetric water content (VWC). The VWC of the treatment group and the control group were 62.0% and 25.0%, respectively. Additionally, the plants were introduced to shade. After three days, we removed the water and shade and placed the plants under natural lighting and cared for them daily. We immediately started collecting flower bud samples while flooding (BDS-SP), then collected buds and inflorescences respectively when the flower buds were about 0.5 cm in diameter (BS) and ligulate flowers were 70% blossomed and tubular flowers were 50% blossomed (FBS) [47].

### 4.3. Measurement of Total Flavonoids

A precisely weighed amount, 0.50 g, of inflorescence powder was placed in a covered conical flask, to which 50 mL of 70% methyl alcohol was added, and was tightly sealed with a stopper. The setup was weighed and ultrasound was applied (power 300 W, frequency 45 kHz) for 40 min, before being chilled and weighed again. The lost weight was replaced with 70% methyl alcohol, shook well, and the subsequent filtrate was obtained. Then, ultraviolet spectroscopy was used to measure the content of total flavonoids. One milliliter of the methanol extract was mixed with 5 mL of distilled water, followed by the addition of 1 mL of 5% (*w*/*w*) NaNO_2_ solution. After 6 and 12 min, 1 and 10 mL, respectively, of 10% (*w*/*w*) Al(NO_3_)_3_ solution and 1 M NaOH were added. The mixture was brought to 25 mL with distilled water and determined at 510 nm using a spectrophotometer [4]. The content of total flavonoids was calculated as rutin equivalents. The linear relationship of rutin was Y = 11.721 X + 0.0012 (R^2^ = 0.9994).

### 4.4. Measurement of Anthocyanins

An accurately weighed amount, 1.00 g, of inflorescence was prepared, to which 10 mL of hydrochloric acid-methanol (1:99, *v*/*v*) solution was added after grinding. Then, it was gently shaken for 24 h at room temperature, centrifuged (Eppendorf Centrifuge 5810 R, Hamburg, Germany) at 12,000 r/min for 10 min, and the supernatant was taken separately. Absorbance was measured at wavelengths of 530 nm and 657 nm. Quantification of anthocyanins was performed using the following equation: Q_Anthocyanins_ = (A530 − 0.25 × A657) × M^−1^, where Q_Anthocyanins_ was the amount of anthocyanins, A530 and A657 were the absorption at the indicated wavelengths and M was the weight of the plant material used for extraction [48].

### 4.5. Analysis of “Hangju” Metabolomics Based on LC-MS Data

The freeze-dried capitula were crushed using a mixer mill (MM 400, Retsch, Shanghai, China, https://www.retsch.cn) with a zirconia bead for 1.5 min at 30 Hz. 100 mg powder was weighed and extracted overnight at 4 °C with 1.0 mL 70% aqueous methanol. Following centrifugation at 10,000 g for 10 min, the extracts were absorbed (CNWBOND Carbon-GCB SPE Cartridge, 250 mg, 3 mL; ANPEL, Shanghai, China, www.anpel.com.cn/cnw) and filtrated (SCAA-104, 0.22 μm pore size; ANPEL, Shanghai, China, http://www.anpel.com.cn) before LC-MS analysis. The quality control sample (QC) was prepared by mixing all of the samples and used to demonstrate the precision of the assay. During the instrumental analysis, a quality control sample was inserted into each of the five test samples to examine the repeatability of the analysis process.

The sample extracts were analyzed using an LC-ESI-MS/MS system (HPLC, Shim-pack UFLC SHIMADZU CBM30A system, www.shimadzu.com.cn; MS, Applied Biosystems 4500 Q TRAP, www.appliedbiosystems.com.cn). The analytical conditions were as follows, HPLC: column, Waters ACQUITY UPLC HSS T3 C18 (1.8 μm, 2.1 mm × 100 mm); solvent system, water (0.04% acetic acid): acetonitrile (0.04% acetic acid); gradient program, 100:0 (*v*/*v*) at 0 min, 5:95 (*v*/*v*) at 11.0 min, 5:95 (*v*/*v*) at 12.0 min, 95:5 (*v*/*v*) at 12.1 min, 95:5 (*v*/*v*) at 15.0 min; flow rate, 0.40 mL/min; temperature, 40 °C; injection volume: 5 μL. The effluent was alternatively connected to an ESI-triple quadrupole-linear ion trap (Q TRAP)-MS.

Linear Ion trap (LIT) and triple quadrupole (QQQ) scans were acquired on a triple quadrupole-linear ion trap mass spectrometer (Q TRAP), API 4500 Q TRAP LC/MS/MS System, equipped with an ESI Turbo Ion-Spray interface, operating in a positive ion mode and controlled using Analyst 1.6 software (AB Sciex, Redwood, CA, USA). The ESI source operation parameters were as follows: ion source, turbo spray; source temperature 550 °C; ion spray voltage (IS) 5500 V; ion source gas I (GSI), gas II(GSII), curtain gas (CUR) were set at 55, 60, and 25.0 psi, respectively; the collision gas (CAD) was high. Instrument tuning and mass calibration were performed with 10 and 100 μmol/L polypropylene glycol solutions in QQQ and LIT modes, respectively. QQQ scans were acquired as MRM experiments with collision gas (nitrogen) set to 5 psi. Declustering Potential (DP) and Collision Energy (CE) for individual MRM transitions was done with further DP and CE optimization. A specific set of MRM transitions were monitored for each period according to the metabolites eluted within this period.

### 4.6. Real-Time Quantitative PCR

Total RNA was extracted from “Hangju” using the TaKaRa MiniBEST Plant RNA Extraction Kit (TaKaRa, Dalian, China) according to the manufacturer’s protocol. cDNA for the real-time qPCR analysis was synthesized from 2 μg of total RNA using the PrimeScript™ RT reagent Kit (TaKaRa, Dalian, China) with gDNA Eraser Perfect Real Time. The Step One Real-Time system (Applied Biosystems, Carlsbad, CA, USA) was used to conduct an amplified reaction consisting of 95 °C for 30 s, followed by 40 cycles of 5 s at 95 °C, and 30 s at 60 °C. The cycle threshold (Ct) value for each PCR reaction was calculated. After completion of the amplification steps, the melting curve was determined for each reaction followed by electrophoresis of the PCR products to confirm the specific amplification of each qPCR. Primer sequences and GenBank accession number are listed in Table S4.

### 4.7. Statistical Analysis

Qualitative and quantitative analysis of metabolites, as well as analysis of metabolite differences and metabolic pathways were referenced from Wang et al. (2018) [49]. The relative expressions were calculated using the 2^−∆∆Cт^ method [50]. Statistical analysis was performed using Microsoft Office Excel 2016 and SPSS 23.0 (IBM Corporation, Armonk, NY, USA).

## Supplementary Materials

Supplementary Materials can be found at https://www.mdpi.com/1420-3049/24/20/3695/s1. Figure S1. The score chart of PLS-DA analysis; Table S1: ALL sample data; Table S2: Metabolites annotated from the KEGG database; Table S3: The result of Venn diagrams; Table S4: Primer sequences and GenBank accession number.

## References

[1] Da Silva J.A.T. (2003). Chrysanthemum: Advances in tissue culture, cryopreservation, postharvest technology, genetics and transgenic biotechnology. Biotechnol. Adv..

[2] Van Der Ploeg A., Heuvelink E. (2015). The influence of temperature on growth and development of chrysanthemum cultivars. J. Hortic. Sci. Biotechnol..

[3] Lin L.-Z., Harnly J.M. (2010). Identification of the phenolic components of chrysanthemum flower (Chrysanthemum morifolium Ramat). Food Chem..

[4] Miao J. (2012). Growth and accumulation of bioactive compounds in medicinal Chrysanthemum morifolium Ramat. cv. ’Chuju’ under different colored shade polyethylene. J. Med. Plants Res..

[5] Chinese Pharmacopoeia Editorial Committee (2015). Pharmacopoeia of the People’s Republic of China.

[6] Ukiya M., Akihisa T., Tokuda H., Suzuki H., Mukainaka T., Ichiishi E., Yasukawa K., Kasahara Y., Nishino H. (2002). Constituents of Compositae plants III. Anti-tumor promoting effects and cytotoxic activity against human cancer cell lines of triterpene diols and triols from edible chrysanthemum flowers. Cancer Lett..

[7] Kim I.S., Koppula S., Park P.-J., Kim E.H., Kim C.G., Choi W.S., Lee K.H., Choi D.-K. (2009). Chrysanthemum morifolium Ramat (CM) extract protects human neuroblastoma SH-SY5Y cells against MPP+-induced cytotoxicity. J. Ethnopharmacol..

[8] Tsuji-Naito K., Saeki H., Hamano M. (2009). Inhibitory effects of Chrysanthemum species extracts on formation of advanced glycation end products. Food Chem..

[9] Lii C.K., Lei Y.P., Yao H.T., Hsieh Y.S., Tsai C.W., Liu K.L., Chen H.W. (2010). Chrysanthemum morifolium Ramat. reduces the oxidized LDL-induced expression of intercellular adhesion molecule-1 and E-selectin in human umbilical vein endothelial cells. J. Ethnopharmacol..

[10] Lee J.S., Kim H.J., Lee Y.S. (2003). A new anti-HIV flavonoid from glucoronide from Chrysanthemum marifolium. Planta Med..

[11] Shao Q.-S., Guo Q.-S., Deng Y.-M., Guo H.-P. (2010). A comparative analysis of genetic diversity in medicinal Chrysanthemum morifolium based on morphology, ISSR and SRAP markers. Biochem. Syst. Ecol..

[12] Zhao W., Zhao J., He L., Sun Y., Cai H. (2013). Molecular structure and the second introns variation of gene F3 ’ H of two medicinal Chrysanthemum morifolium populations. Biochem. Syst. Ecol..

[13] Huang L., Guo L., Ma C., Gao W., Yuan Q. (2011). Top-geoherbs of traditional Chinese medicine: Common traits, quality characteristics and formation. Front. Med..

[14] Wang T., Zhu Z., Guo Q., Mao P. (2013). Variation in major flavonoids glycosides and caffeoylquinic acids during florescence of three Chrysanthemum morifolium Ramat cv. ’Hangju’ genotypes. Biochem. Syst. Ecol..

[15] Ueyama Y., Suzuki K., Fukuchi-Mizutani M., Fukui Y., Miyazaki K., Ohkawa H., Kusumi T., Tanaka Y. (2002). Molecular and biochemical characterization of torenia flavonoid 3′-hydroxylase and flavone synthase II and modification of flower color by modulating the expression of these genes. Plant. Sci..

[16] Harborne J.B., Williams C.A. (2000). Advances in flavonoid research since 1992. Phytochemistry.

[17] Kumar A., Singh B., Singh K. (2015). Functional characterization of flavanone 3-hydroxylase gene from Phyllanthus emblica (L.). J. Plant. Biochem. Biotechnol..

[18] Pawlikowska-Pawlega B., Gruszecki W.I., Misiak L., Paduch R., Piersiak T., Zarzyka B., Pawelec J., Gawron A. (2007). Modification of membranes by quercetin, a naturally occurring flavonoid, via its incorporation in the polar head group. Biochim. Biophys. Acta..

[19] Netzel M., Strass G., Kaul C., Bitsch I., Dietrich H., Bitsch R. (2002). In vivo antioxidative capacity of a composite berry juice. Food Res. Int..

[20] Ashida H., Fukuda I., Yamashita T., Kanazawa K. (2000). Flavones and flavonols at dietary levels inhibit a transformation of aryl hydrocarbon receptor induced by dioxin. Febs. Lett..

[21] Deavours B.E., Dixon R.A. (2005). Metabolic engineering of isoflavonoid biosynthesis in alfalfa. Plant. Physiol..

[22] Huang L.-Q., Guo L.-P. (2007). Secondary metabolites accumulating and geoherbs formation under enviromental stress. China J. Chin. Mater. Med..

[23] Zou Q.-J., Wang T., Guo Q.-S., Xiao Y.-M., Wu L.-W. (2018). Cloning and expression analysis of F3’H and quantification of downstream products in Chrysanthemum morifolium under flooding stress. China J. Chin. Mater. Med..

[24] Jackson M.B., Colmer T.D. (2005). Response and adaptation by plants to flooding stress. Ann. Bot.

[25] Chen W., Gong L., Guo Z., Wang W., Zhang H., Liu X., Yu S., Xiong L., Luo J. (2013). A novel integrated method for large-scale detection, identification, and quantification of widely targeted metabolites: Application in the study of rice metabolomics. Mol. Plant..

[26] Fraga C.G., Clowers B.H., Moore R.J., Zink E.M. (2010). Signature-Discovery Approach for Sample Matching of a Nerve-Agent Precursor Using Liquid Chromatography-Mass Spectrometry, XCMS, and Chemometrics. Anal. Chem..

[27] Brennan L. (2017). The nutritional metabolomics crossroads: How to ensure success for dietary biomarkers. Am. J. Clin. Nutr..

[28] Mallick S., Singh S.K., Sarkar C., Saha B., Bhadra R. (2005). Human placental lipid induces melanogenesis by increasing the expression of tyrosinase and its related proteins in vitro. Pigment. Cell Res..

[29] Kanu P.J., Zhu K., Kanu J.B., Zhou H., Qian H., Zhu K. (2007). Biologically active components and nutraceuticals in sesame and related products: A review and prospect. Trends Food Sci. Technol..

[30] Bose U., Hewavitharana A.K., Ng Y.K., Shaw P.N., Fuerst J.A., Hodson M.P. (2015). LC-MS-Based Metabolomics Study of Marine Bacterial Secondary Metabolite and Antibiotic Production in Salinispora arenicola. Mar. Drugs.

[31] Saito K., Matsuda F. (2010). Metabolomics for Functional Genomics, Systems Biology, and Biotechnology. Annu. Rev. Plant Biol..

[32] Duan L.-X., Chen T.-L., Li M., Chen M., Zhou Y.-Q., Cui G.-H., Zhao A.-H., Jia W., Huang L.-Q., Qi X. (2012). Use of the Metabolomics Approach to Characterize Chinese Medicinal Material Huangqi. Mol. Plant..

[33] Kueger S., Steinhauser D., Willmitzer L., Giavalisco P. (2012). High-resolution plant metabolomics: From mass spectral features to metabolites and from whole-cell analysis to subcellular metabolite distributions. Plant. J..

[34] Wang D., Zhang L., Huang X., Wang X., Yang R., Mao J., Wang X., Wang X., Zhang Q., Li P. (2018). Identification of Nutritional Components in Black Sesame Determined by Widely Targeted Metabolomics and Traditional Chinese Medicines. Molecules.

[35] Wang X., Li C., Zhou C., Li J., Zhang Y. (2017). Molecular characterization of the C-glucosylation for puerarin biosynthesis in Pueraria lobata. Plant. J..

[36] Meng J., Wang B., He G., Wang Y., Tang X., Wang S., Ma Y., Fu C., Chai G., Zhou G. (2019). Metabolomics Integrated with Transcriptomics Reveals Redirection of the Phenylpropanoids Metabolic Flux in Ginkgo biloba. J. Agric. Food Chem.

[37] Mattson M.P. (2008). Hormesis defined. Ageing Res. Rev..

[38] Calabrese E.J., Baldwin L.A. (1998). Hormesis as a biological hypothesis. Environ. Health Perspect..

[39] Guo L., Zhang X., Yang G., Huang L., Ma J. (2011). Hormesis and its application in medicinal plant growing. China J. Chin. Mater. Med..

[40] Liu S.P., Bi Y.T., Tian W., Xue Y.H. (2013). Comparative Physiological Responses of Two *Buxus* Species on Flooding Stress. Appl. Mech. Mater..

[41] Guo L.-P., Huang L.-Q., Yan H., Lv D.-M., Jiang Y.-X. (2005). Habitat characteristics for the growth of Atractylodes lancea based on GIS. China J. Chin. Mater. Med..

[42] Cipollini M.L., Paulk E., Cipollini D.F. (2002). Effect of nitrogen and water treatment on leaf chemistry in horsenettle (Solanum carolinense), and relationship to herbivory by flea beetles (Epitrix spp.) and tobacco hornworm (Manduca sexta). J. Chem. Ecol..

[43] Winkel-Shirley B. (2002). Biosynthesis of flavonoids and effects of stress. Curr. Opin. Plant. Biol..

[44] Halliwell B., Rafter J., Jenner A. (2005). Health promotion by flavonoids, tocopherols, tocotrienols, and other phenols: Direct or indirect effects? Antioxidant or not?. Am. J. Clin. Nutr..

[45] Wang L., Lee I.M., Zhang S.M., Blumberg J.B., Buring J.E., Sesso H.D. (2009). Dietary intake of selected flavonols, flavones, and flavonoid-rich foods and risk of cancer in middle-aged and older women. Am. J. Clin. Nutr..

[46] Jan A.T., Kamli M.R., Murtaza I., Singh J.B., Ali A., Haq Q.M.R. (2010). Dietary Flavonoid Quercetin and Associated Health BenefitsAn Overview. Food Rev. Int..

[47] Wang T., Guo Q.-s., Mao P.-F. (2014). Flavonoid accumulation during florescence in three Chrysanthemum morifolium Ramat cv. ’Hangju’ genotypes. Biochem. Syst. Ecol..

[48] Mehrtens F., Kranz H., Bednarek P., Weisshaar B. (2005). The Arabidopsis transcription factor MYB12 is a flavonol-specific regulator of phenylpropanoid biosynthesis. Plant. Physiol..

[49] Wang Y., Lysoe E., Armarego-Marriott T., Erban A., Paruch L., van Eerde A., Bock R., Liu-Clarke J. (2018). Transcriptome and metabolome analyses provide insights into root and root-released organic anion responses to phosphorus deficiency in oat. J. Exp. Bot..

[50] Vandesompele J., De Preter K., Pattyn F., Poppe B., Van Roy N., De Paepe A., Speleman F. (2002). Accurate normalization of real-time quantitative RT-PCR data by geometric averaging of multiple internal control genes. Genome Biol..

