# Supplementary figures and images for: Widely Targeted Metabolomics Analysis Reveals the Effect of Flooding Stress on the Synthesis of Flavonoids in Chrysanthemum morifolium
Source: Molecules. 2019 Oct 14;24(20):3695. doi: 10.3390/molecules24203695 (PMC6832227; doi:10.3390/molecules24203695)

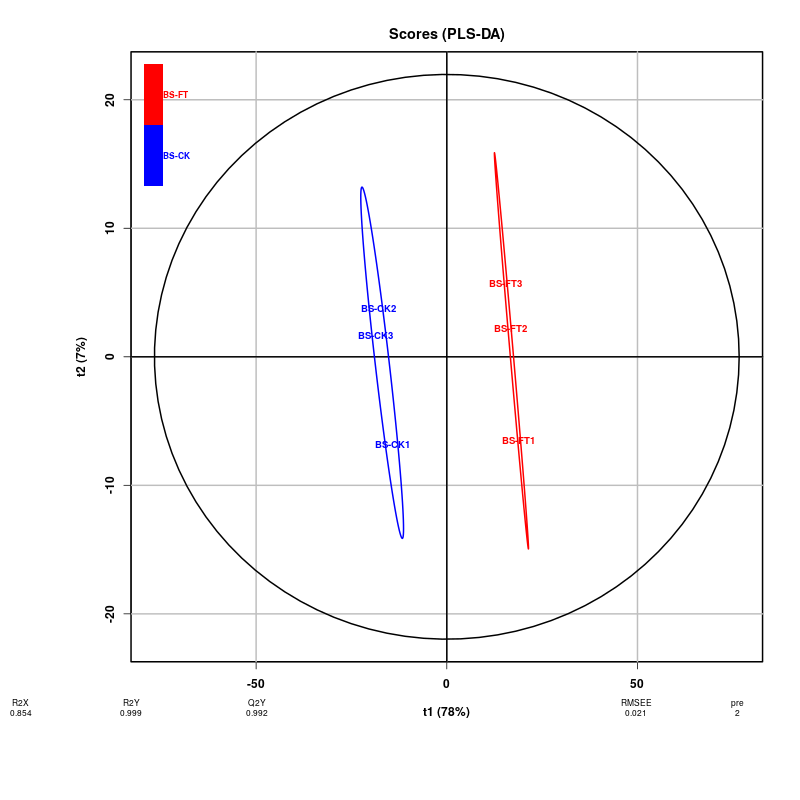

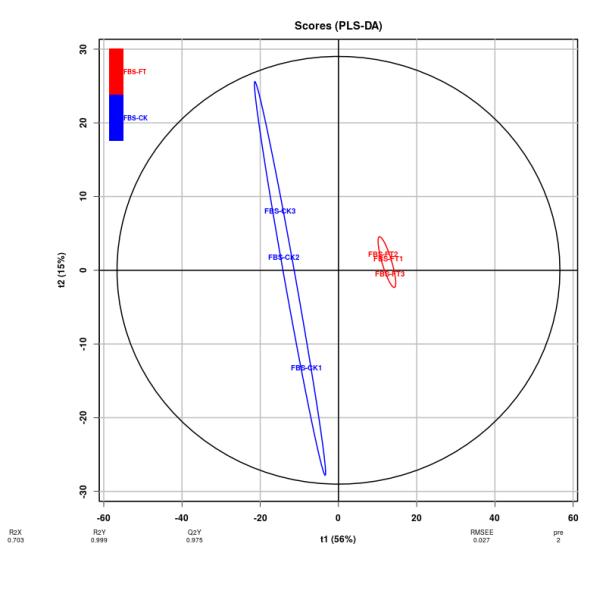


Figure S1. The score chart of PLS-DA analysis

Supplement: Supplementary file 1 [file molecules-24-03695-s001.zip › supplementary materials/Figure S1.docx]
